# Evaluation of resistance to pyrethroid and organophosphate adulticides and *kdr* genotyping in *Aedes aegypti* populations from Roraima, the northernmost Brazilian State

**DOI:** 10.1186/s13071-020-04127-w

**Published:** 2020-05-20

**Authors:** Ramão Luciano Nogueira Hayd, Luana Carrara, Joel de Melo Lima, Nathalia Coelho Vargas de Almeida, José Bento Pereira Lima, Ademir Jesus Martins

**Affiliations:** 1 Laboratório de Parasitologia e Monitoramento de Artrópodes Vetores da Amazônia, Universidade Federal de Roraima, Boa Vista, RR Brazil; 2 Laboratório de Fisiologia e Controle de Artrópodes Vetores, Instituto Oswaldo Cruz, FIOCRUZ, Av Brasil 4365, Manguinhos, Rio de Janeiro, RJ Brazil; 3 Núcleo de Febre Amarela e Dengue, Coordenadoria Geral de Vigilância em Saúde, Secretaria de Estado da Saúde de Roraima, Boa Vista, RR Brazil; 4 Núcleo de Estadual de Entomologia, Coordenadoria Geral de Vigilância em Saúde, Secretaria de Estado da Saúde de Roraima, Boa Vista, RR Brazil; 5 Instituto Nacional de Ciência e Tecnologia em Entomologia Molecular, INCT-EM, Universidade Federal do Rio de Janeiro, Rio de Janeiro, RJ Brazil

**Keywords:** Vector control, Insecticide resistance monitoring, Rorainópolis, Pacaraima, Bonfim, Boa Vista, Deltamethrin, Malathion

## Abstract

**Background:**

Roraima, the northernmost State in Brazil, borders Venezuela and Guyana. Although mostly covered by the tropical forests, the urban centers of this state are highly infested with *Ae. aegypti* and are endemic for dengue, Zika and chikungunya. We accessed the insecticide resistance status of *Ae. aegypti* populations from the capital Boa Vista, two cities on international borders (Pacaraima and Bonfim) and Rorainópolis bordering Amazonas State, in order to evaluate the chemical control efficacy in these localities.

**Methods:**

Tests with World Health Organization (WHO)-like tubes impregnated with the pyrethroid deltamethrin (0.05% and 0.12%) and the organophosphate malathion (0.7%) were conducted with *Ae. aegypti* from Boa Vista, Pacaraima, Bonfim and Rorainópolis, collected in 2016 and 2018. Genotyping of *kdr* mutations, related to resistance to pyrethroids, was performed for the SNP variations at sites 1016 and 1534 of the voltage gated sodium channel gene (*Na*_*V*_) with a TaqMan qPCR approach.

**Results:**

*Aedes albopictus* was absent in our collections, and therefore only *Ae. aegypti* was tested. All *Ae. aegypti* populations were susceptible to 0.7% malathion in 2016; however, mortality dropped to under 90% in Bonfim and Pacaraima populations in 2018. All populations were resistant to 0.05% deltamethrin in both years. The time that 50% of females suffered knockdown (*Kd*T_50_) under exposure to 0.05% deltamethrin was 3.3–5.9-fold longer in mosquitoes from the natural populations compared to the susceptible Rockefeller strain. Only the Pacaraima population (2018) remained resistant to 0.12% deltamethrin. *Kdr* genotyping revealed the absence of the wild-type Na_V_S haplotype (1016Val + 1534Phe) in the populations from Roraima, indicating that all tested insects had a genetic background for pyrethroid resistance. The double *kdr* Na_V_R2 haplotype (1016Ile + 15434Cys) was present in higher frequencies in all populations except for Rorainópolis, where this haplotype seems to have arrived recently.

**Conclusions:**

These results are important for the knowledge about insecticide resistance status of *Ae. aegypti* populations from Roraima and will help improve vector control strategies that may be applied to diverse localities under similar geographical and urban conditions.
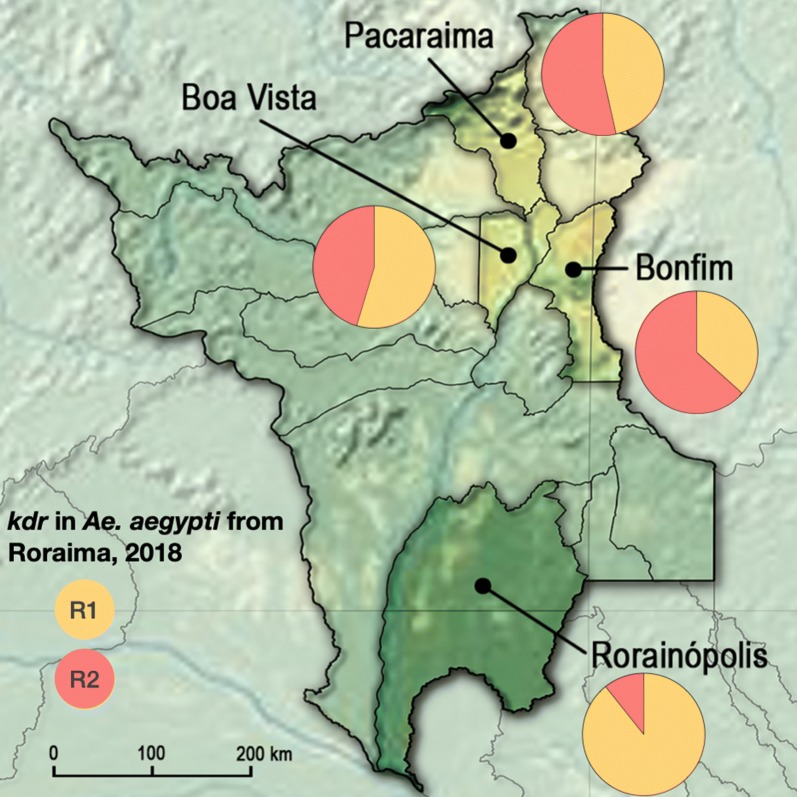

## Background

The globalization and rapid adaptation of *Aedes aegypti* to urban conditions favor its dispersion around the globe, posing a serious threat to human public health since this mosquito is a vector of many arboviruses such as dengue, Zika, chikungunya and urban yellow fever, among others [1]. Roraima is a state in the extreme North of Brazil bordering Venezuela and Guyana and is therefore an important Brazilian doorway for the entrance of emergent and re-emergent arbovirus, given the circulation of wildlife, people and goods between the borders. In addition, population genetic studies have suggested that the re-infestation of *Ae. aegypti* in Brazil likely occurred from Venezuela during the 1970’s [2], evidencing the need of constant entomological surveillance in Roraima State.

In 2010, the re-emergence of DENV-4 in Brazil, with a hypothesized origin in Venezuela, was first recorded in Boa Vista, the capital of Roraima, rapidly spreading to other states in the country: Amazonas and Pará (North); Bahia, Pernambuco and Piauí (Northeast); and Rio de Janeiro and São Paulo (Southeast) [3]. Attempting to block DENV-4 circulation, efforts targeted the elimination of larval breeding sites and the intensification of chemical control with employment of the insect growth regulator diflubenzuron and the pyrethroid adulticide deltamethrin. However, this strategy did not significantly reduce *Ae. aegypti* infestation and rapidly increased the levels of insecticide resistance to the pyrethroid [4].

Insecticide resistance (IR) is a threat to the control of arboviruses at a global scale [5]. In Brazil, a nationwide IR monitoring programme coordinated by the Ministry of Health (MoH) has been screening the status of susceptibility to recommended chemicals in *Ae. aegypti* populations of strategic localities since 1999 [6]. In previous surveys, pyrethoid resistance was detected in *Ae. aegypti* from Boa Vista and Pacaraima [7, 8], probably due to selection of *kdr* mutations. These mutations are single nucleotide polymorphisms (SNPs) in the voltage gated sodium channel gene (*Na*_*V*_), which encodes a neuronal transmembrane protein that is the target of pyrethroids and DDT. In *Ae. aegypti* populations from Brazil, at least four principal SNPs in the *Na*_*V*_ gene (V410L, I1011M, V1016I and F1534C) have been identified [8–10], whereas the latter two were more related to resistance to the pyrethroid knockdown effect (*kdr*) [10]. All *Ae. aegypti* samples evaluated from Boa Vista and Pacaraima in 2011 harbored *kdr* mutations, in either Na_V_R1 (1016Val + 1534Cys^*kdr*^) or Na_v_R2 (1016Ile^*kdr*^ + 1534Cys^*kdr*^) haplotypes [8]. In addition to the presence of *kdr* mutations, alterations in the activity of enzymes related to metabolic resistance, such as glutathione S-tranferases (GSTs) and esterases, were also observed in these populations [7]. Given resistance to pyrethroids detected in *Ae. aegypti* populations throughout the country, the MoH has replaced these compounds with the organophosphate malathion [7], which in Roraima State was introduced in 2015 by governmental campaigns. Pyrethroids however habitually continued being applied through household sprays vastly available in market stores and by governmental programmes against malaria vectors in endemic regions.

Herein, we evaluate the profile of resistance to pyrethroid and organophosphate adulticides in *Ae. aegypti* populations of four important cities of Roraima State, Brazil, in 2016 and 2018, extending the analyses to localities not previously assessed. *Kdr* haplotype frequency was also investigated.

## Methods

### Collections and laboratory rearing conditions

**Fig. 1:**
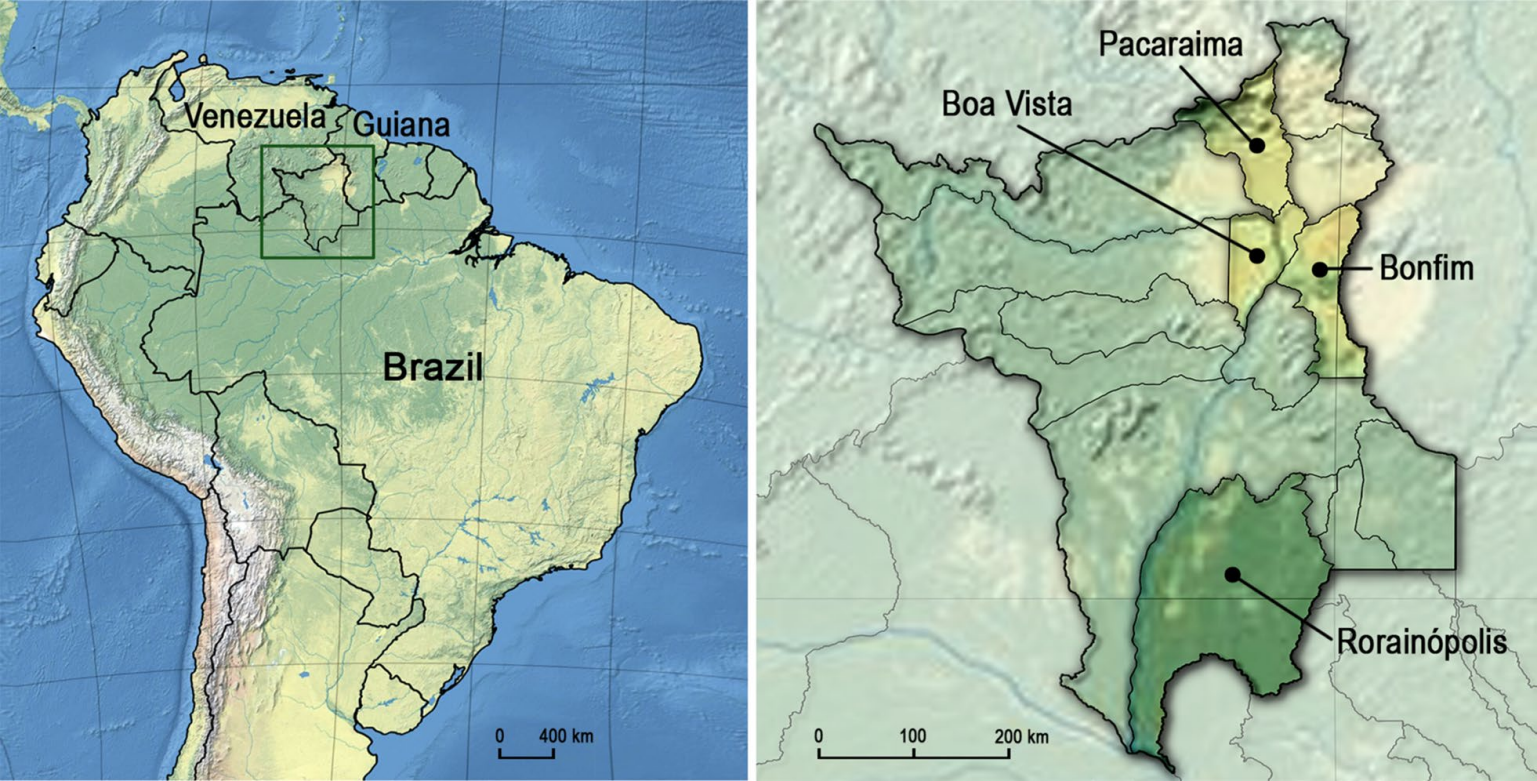
Map showing the location of Roraima, the northernmost Brazilian state, and its borders with Venezuela and Guyana (left). Insecticide resistance studies were carried out in 2016 and 2018 on *Aedes aegypti* populations from Boa Vista, Bonfim, Pacaraima and Rorainópolis (right)

Roraima State has a territory of 224,273.83 km^2^ and is the least populous Brazilian state with only 0.29% of the total population in the country (605,761 inhabitants as stipulated by the Brazilian Institute of Geography and Statistics, in 2019) [11]. Roraima has limits with the Amazon in the South and borders Venezuela and Guyana (Fig. 1). The capital, Boa Vista, is 741.1 km from Manaus (the capital of Amazonas State) by the BR-174 road. This same highway connects Boa Vista and Pacaraima on the border of Venezuela, 2013.5 km to the North.

Collections of mosquito eggs were performed in four of the most important cities of Roraima State, including the capital, Boa Vista (02° 49′ 11″ N, 60° 40′ 24″ W), Bonfim (02° 45′ 22.25″ N, 60° 7′ 6.53″ W) in the Northeast on the border with Guyana, Pacaraima (04° 25′ 01″ N, 61° 08′ 27″ W) in the North on the border with Venezuela and Rorainópolis in the South on the Amazonas State limits (00° 56′ 22.62″ N, 60° 26′ 21.91″ W). Ovitraps consisted of 800 ml black plastic cups, containing 300 ml of a 0.04% yeast extract solution to attract gravid females which would lay their eggs in a wood paddle immersed in this solution for 5–7 days. The total of 150 traps were installed in Boa Vista and 50 in each of the other cities, following standard recommendations [12]. Paddles were shipped to the laboratory Núcleo de Pesquisa Observatório da Saúde at the Federal University of Roraima State (UFRR), eggs were stimulated to hatch and larvae reared under laboratory conditions as described elsewhere [12]. Adult mosquitoes were screened for species identification and maintained in cardboard cylindrical cages (17 × 18 cm) with a 10% sucrose solution w/v offered *ad libitum*. Anesthetized Wistar rats (*Rattus norvegicus*) were offered to blood feed females in order to produce eggs of an F1 generation, following procedures as recommended in the license # 12/2015 approved by the ethical committee on animal use of UFRR (CEUA-UFRR). The reference lineage Rockefeller *Ae. aegypti* mosquitoes, maintained at Laficave/Fiocruz since 1999 [12], were raised in parallel and adopted in all assays as a control of insecticide susceptibility and vigor under test conditions.

### Insecticide resistance related assays

#### Bioassays

Bioassays were performed with F1 generation *Ae. aegypti* females 3–5 days post-adult emergence with World Health Organization (WHO) tube tests [13]. We employed the pyrethroid deltamethrin (technical grade; Sigma-Aldrich, St. Louis, USA) dissolved in acetone to a 1% stock solution, this was then diluted to a concentration of 0.05% and 0.12% in silicone oil (Dow Corning, Midland, USA) as the solvent carrier. Malathion (Sangrose Agroquímica, Curitiba, Brazil) emulsion concentrate was diluted in the silicone oil to a concentration of 0.7% and subsequently applied over a 12 × 15 cm filter paper (Whatman Grade 1) as previously described [14]. It is noteworthy that olive oil is the solvent/carrier recommended for paper impregnation with organophosphates. However, we employed silicone oil since we did not have a source of olive oil (chemical grade) with a certificate of analyses available in the country. Mosquitoes were exposed for 1 h in the tube chamber with insecticide and then gently transferred to the resting chamber until mortality was registered 24 h later. In addition, the knockdown rate was evaluated every 5 min (or 2 min for the Rockefeller strain) during exposure to 0.05% deltamethrin. A probit analysis [15] was incorporated in order to calculate the time when 50% of females were knocked down (*Kd*T_50_). The knockdown resistance ratios (RR *Kd*T_50_) of the populations were obtained by the quotient between the *Kd*T_50_ of the populations with the Rockefeller strain, the control strain.

#### kdr genotyping

DNA was extracted from male mosquitoes and genotyped for the single nucleotide polymorphisms (SNPs) at the 1016 (Val^+^ or Ile^*kdr*^) and 1534 (Phe^+^ or Cys^*kdr*^) sites of the voltage gated sodium channel gene (*Na*_*V*_) using exactly the same procedures and reagents as described by Macoris et al. [16]. As these sites are linked in the same gene, both SNPs were considered in order to define the genotype frequencies for a single locus [8]. The haplotypes usually found in *Ae. aegypti* populations from Brazil are the wild-type Na_V_S (1016 Val + 1534 Phe) and the *kdr*s Na_V_R1 (1016Val + 1534Cys) and Na_V_R2 (1016Ile + 1534Cys), whereas the possible genotypes are SS, SR1, SR2, R1R1, R1R2 and R2R2. As *kdr* mutations act as a recessive trait, insects resistant to pyrethroids based on this target site mechanism present one of the genotypes R1R1, R1R2 or R2R2. The Na_V_R2 haplotype generates higher levels of resistance [14].

## Results

### Bioassays

We evaluated the susceptibility status of *Ae. aegypti* from four Roraima municipalities to the pyrethroid deltamethrin (0.05% and 0.12%) and the organophosphate malathion (0.7%) adulticides, in 2016 and 2018.

**Fig. 2:**
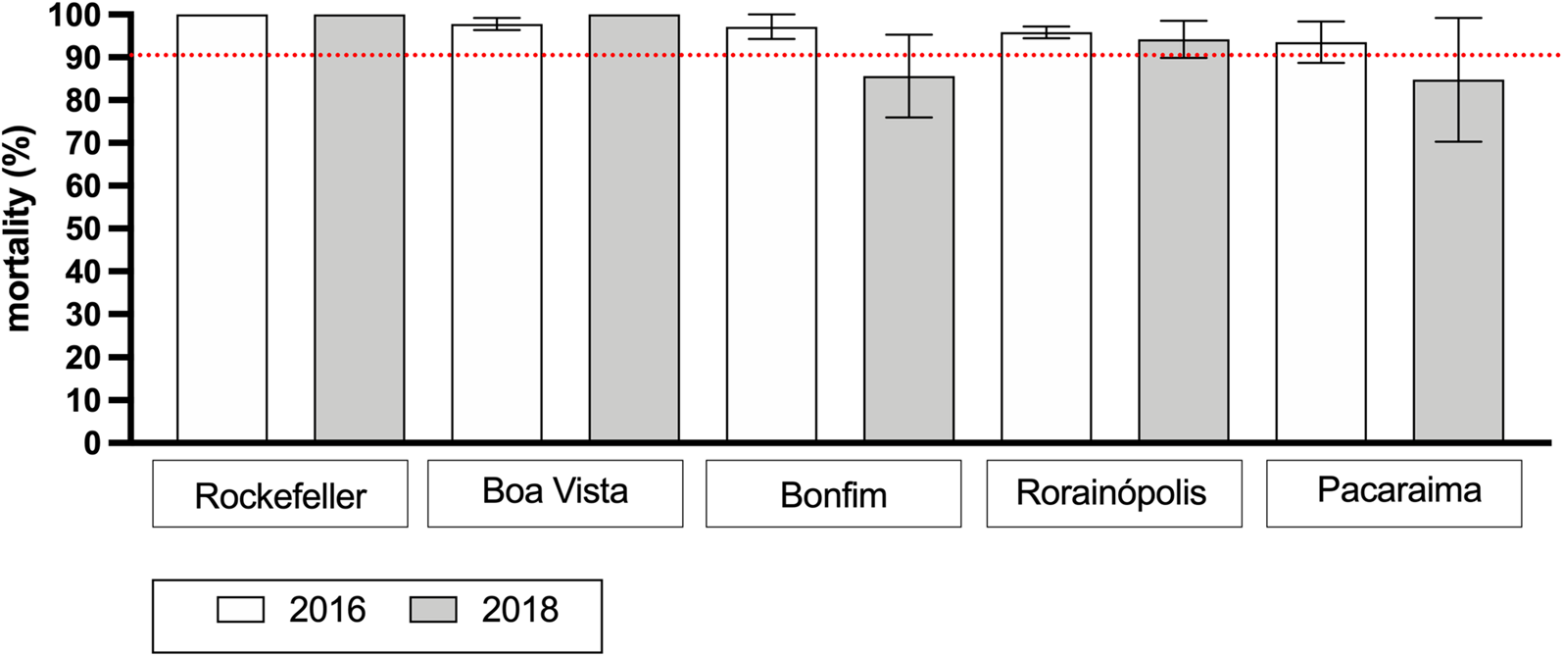
Evaluation of mortality of *Ae. aegypti* from Roraima caused by the organophosphate malathion. Bars indicate the mean percent mortality (± standard error of the mean) registered 24 h after exposure to malathion 0.7% for 1 h. Populations with mortality > 90% (red dotted line) are classified as resistant to the insecticide

In 2016, populations exhibited some survival to malathion 0.7%, however with mortality rates greater than 90%, except that from Rorainópolis, attaining a mortality rate of 100% (Fig. 2). There was a reduction in the mortality levels in 2018 in all localities, ranging from 84.8% in Pacaraima to 94.2% in Rorainópolis (Table 1). Therefore, Pacaraima and Boa Vista were classified as resistant to malathion in 2018.

**Table 1:** Bioassays with the organophosphate malathion (0.7%) in *Aedes aegypti* populations from Roraima State, Brazil (2016 and 2018)

Population	2016		2018	
	*n*	Mean mortality ± SD (%)	*n*	Mean mortality ± SD (%)
Boa Vista	175	97.8 ± 2.48	197	100 ± 0
Bonfim	171	97.1 ± 4.97	181	85.6 ± 16.77
Rorainópolis	185	95.9 ± 2.37	184	94.2 ± 7.51
Pacaraima	188	93.6 ± 8.37	178	84.8 ± 25.01

*Abbreviations*: n, total number of insects used/ population; SD, standard deviation

**Fig. 3:**
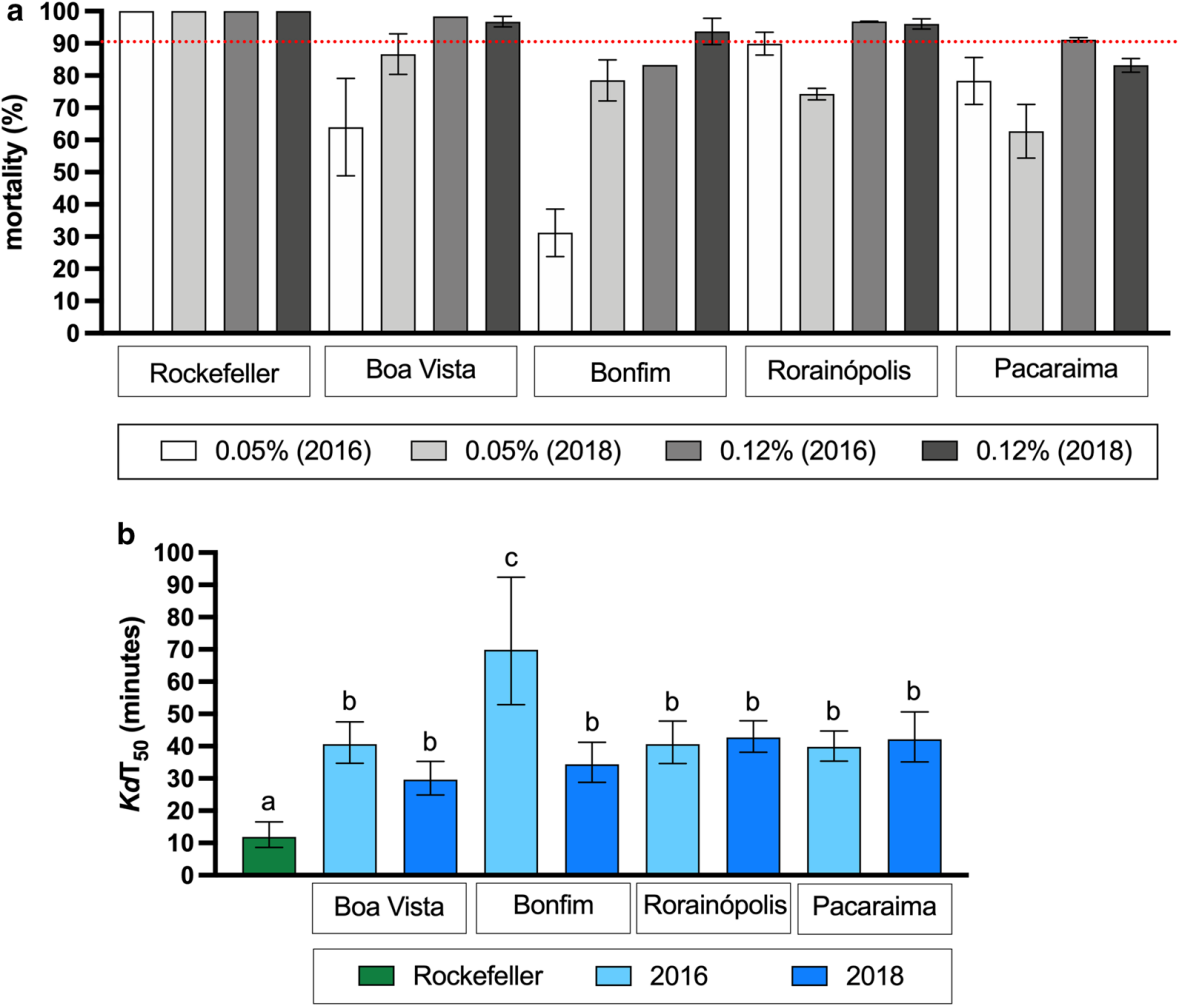
Evaluation of mortality of *Ae. aegypti* from Roraima caused by the pyrethroid deltamethrin. **a** Bars indicate the mean percent mortality (± standard error of mean) registered 24 h after 1 h exposure to deltamethrin. Populations with mortality beyond 90% (red dotted line) are classified as resistant to the insecticide. **b***Kd*T_50_ with 95% CI. Bars with identical letters indicate similar times (overlapped 95% CI)

With regard to the pyrethroid, all populations were considered resistant both in 2016 and 2018 when evaluated with 0.05% deltamethrin. There was an increase from 2016 to 2018 in the mortality in Boa Vista (64.0 to 86.6%) and Bonfim (31.2 to 78.5%) and a decrease in Rorainópolis (89.9 to 74.3%) and Pacaraima (78.3 to 61.7%). A higher dosage of deltamethrin (0.12%) was tested in parallel, and as expected, this higher concentration increased the mortality in all cases; however, mortality did not reach 100% in any population (Fig. 3a). We also evaluated the knockdown rate during exposure to deltamethrin 0.05% for 1h (Additional file 1: Figure S1). According to a probit analysis, 50% of the Rockefeller strain females were knocked down (*Kd*T_50_) within 11.9 min, compared to 29.6 and 69.9 min in Boa Vista (2018) and Bonfim (2016) populations, respectively (Table 2). Nevertheless, the *Kd*T_50_ values of all populations were similar if we consider the 95% confidence (CI) interval, except for Bonfim population in 2016, which showed a higher *Kd*T_50_ (Fig. 3b). The knockdown-resistance ratio (*Kd*T-RR_50_) of the populations ranged from 2.5 to 5.9 (Table 2).

**Table 2:** Time of knockdown to the pyrethroid deltamethrin (0.05%) in *Aedes aegypti* populations from Roraima State, Brazil (2016 and 2018)

Populations	2016				2018			
	*n*	*Kd*T_50_ min	95% CI	*Kd*T-RR_50_	*n*	*Kd*T_50_ min	95% CI	*Kd*T-RR_50_
Boa Vista	320	40.6	34.7–47.5	3.4	178	29.6	24.9–35.3	2.5
Bonfim	146	69.9	52.9–92.4	5.9	172	34.4	28.8–41.2	2.9
Rorainópolis	187	40.6	34.6–47.8	3.4	177	42.7	38.1–47.9	3.6
Pacaraima	182	39.8	35.4–44.7	3.3	193	42.1	35.1–50.6	3.5
Rockefeller strain	182	11.9	8.6–16.5					

*Abbreviations*: n, total number of insects used/ population; 95% CI, 95% confidence interval

### *kdr* genotyping

**Fig. 4:**
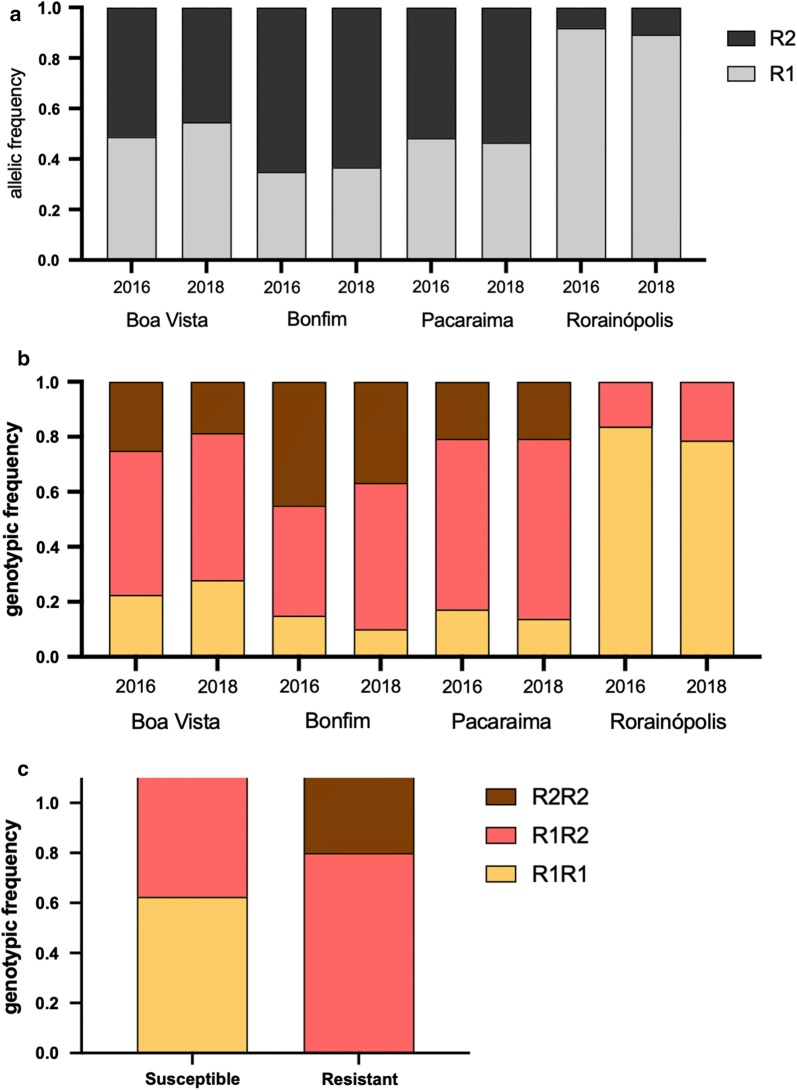
Genotyping of *kdr* mutations in *Ae. aegypti* populations from Roraima. **a** Allelic frequencies of the *kdr* haplotypes. **b** Genotypic frequencies. **c** Genotypic frequencies of *Ae. aegypti* from Boa Vista and Bonfim (2016) susceptible resistant to deltamethrin 0.05%

We successfully genotyped 282 *Ae. aegypti* mosquitoes from Roraima State for both 1016 (Val + or Ile^*kdr*^) and 1534 (Phe^+^ or Cys^*kdr*^) sites. All tested insects demonstrated a *kdr* mutation in at least one site, presenting genotypes: homozygotes for the 1534 *kdr* (R1R1) and the double *kdr* 1016 + 1534 (R2R2) in addition to the heterozygote (R1R2). Therefore, there was no evidence of the wild-type haplotype S (1016Val + 1534Phe) in *Ae. aegypti* populations from Roraima (Fig. 4a).

The double *kdr* haplotype R2 (1016 Ile + 1534Cys) had a higher frequency than R1 (1016 Val + 1534Cys) in Boa Vista (2018), Bonfim (2016 and 2018) and Pacaraima (2016 and 2018) (Table 3). In Rorainópolis, R2 showed low frequency (8 and 11% in 2016 and 2018, respectively), only appearing in heterozygotes R1R2 (Fig. 4b). In all cases, the genotypic frequencies did not deviate from the Hardy-Weinberg equilibrium assumption (Table 3).

**Table 3:** Frequencies of *kdr* genotypes in *Aedes aegypti* populations from Roraima State, Brazil (2016 and 2018)

Locality	Year	*n*	Genotypic frequency	Allelic frequency	HWE test^a^					
			R1R1	R1R2	R2R2	S	R1	R2	c2	*P*
Boa Vista	2016	40	0.225	0.525	0.250	0	0.488	0.513	0.1	0.749
	2018	43	0.279	0.535	0.186	0	0.547	0.453	0.3	0.604
Bonfim	2016	40	0.150	0.400	0.450	0	0.350	0.650	0.6	0.445
	2018	30	0.100	0.533	0.367	0	0.367	0.633	0.7	0.417
Rorainópolis	2016	43	0.837	0.163	0	0	0.919	0.081	0.3	0.561
	2018	28	0.786	0.214	0	0	0.893	0.107	0.4	0.525
Pacaraima	2016	29	0.172	0.621	0.207	0	0.483	0.517	0.1	0.749
	2018	29	0.138	0.655	0.207	0	0.466	0.534	0.3	0.604

^a^Hardy-Weinberg equilibrium test, with probability considering the Chi-squared distribution for 1 degree of freedom

*Abbreviation*: n, total number of evaluated samples for both 1016 and 1534 SNPs

When comparing the genotypic frequencies between the 2016 and 2018 collections, the R2R2 genotype decreased in Boa Vista (25 to 18.6%), Bonfim (45 to 36.7%) and particularly in Pacaraima (45.5 to 20.7%). Indeed, as aforementioned, the levels of mortality to 0.05% deltamethrin increased in both Boa Vista and Bonfim, however decreasing in Pacaraima (Fig. 3b). We also genotyped some dead (susceptible) and survived (resistant) mosquitoes from Boa Vista and Bonfim 24 h after 1 h of exposure to 0.05% deltamethrin. Although the number of samples was small (41 live and 14 dead), the R1R1 genotype was absent in the resistant group while R2R2 was more representative (Fig. 4c).

## Discussion

Here, we showed that *Ae. aegypti* populations from Roraima State, Brazil collected in 2016 and 2018 were resistant to the pyrethroid deltamethrin and under the process of becoming resistant to the organophosphate malathion. Remarkably, only *Ae. aegypti* was present in these collections, regardless of a recent register of *Ae. albopictus* in a rural area of Rorainopolis.

Pyrethroid resistance in *Ae. aegypti* from Roraima State had already been high in previous evaluations. The first registers were in 2007 and 2010 when populations of *Ae. aegypti* from the capital Boa Vista were detected as pyrethroid-resistant [4, 17]. In 2011, the population from Pacaraima presented the second highest resistance ratio (RR_95_ = 60.3) in the whole country [7]. Herein we demonstrate that even two years after pyrethroid governmental application was substituted by malathion, pyrethroid resistance has been maintained in *Ae. aegypti* populations from the four localities evaluated. It is noteworthy that a new diagnostic dose was established for deltamethrin (0.03%) in WHO paper tests [13]. In this study we adopted a higher dosage (0.05%), signifying that the rate of mortality would probably be even lower if tested with 0.03% deltamethrin papers.

The Brazilian Ministry of Health started replacing pyrethroids with the organophosphate malathion in ultra-low volume-based applications against *Ae. aegypti* in 2010 [7], although pyrethroids are still used in campaigns against other vectors such as anophelines, phlebotomines and triatomines. In Roraima State, the first stock of malathion was received in December 2015. Herein, we showed that all four *Ae. aegypti* populations collected in 2016, although not 100% killed by malathion 0.7%, had mortality above 90%, and were not classified as resistant. The rate of mortality decreased to under 90% in Bonfim and Pacaraima two years later, the mosquitoes therefore being classified as resistant to malathion. The diagnostic dose for malathion indicated by the WHO is a bit higher, 0.8% [13]. Had we used 0.8% instead of 0.7%, the decrease in the mortality levels from 2016 to 2018 might have also been noticeable.

Different from what occurs with pyrethroids, the active ingredient of household sprays and other governmental campaigns, the only probable source of organophosphate pressure was the governmental campaigns with malathion at that time. The larvicide temephos has in theory not been applied in Roraima since 2013, when it was definitively substituted by IGRs [7]. The most recent data about temephos resistance in Roraima indicated resistance ratios (RR_50_) of 2.0 in Boa Vista (2010) and 4.3 in Pacaraima (2011), which were not considered high levels of resistance [4, 17]. However, we cannot reject the possibility that mechanisms prior selected by temephos and pyrethroids are inducing cross-resistance to malathion, as reported in some classical studies. For instance, a laboratory strain of *Culex quinquefasciatus* selected for temephos resistance in larvae developed cross-resistance to several organophosphate adulticides, including malathion [18]. On the other hand, in Guadeloupe and Saint Martin Caribbean islands, *Ae. aegypti* populations developed high levels of resistance to temephos (8.9–33.1-fold) but low levels to malathion (1.7–4.4-fold) [19].

Concerning the possible mechanisms selected for insecticide resistance, alterations in the activity of GST and esterase enzymes were detected in Boa Vista (2007) and Pacaraima (2011). Reduced activity of the acetylcholinesterase enzyme was also observed in Pacaraima (2011) [7]. Lineages of *Ae. aegypti* Brazilian populations that acquired resistance to malathion through selection pressure in the laboratory exhibited increased activity of GST, multi-function oxidases (MFO P450) and esterases, as determined by biochemical analyses [20]. Overexpression of genes related to metabolic resistance was detected in *Ae. aegypti* populations from the Caribbean, such as French Guyana and French West Indies islands [19, 21]. On the other hand, the high levels of resistance to pyrethroids in Roraima might be partially justified by the absence of the wild-type Na_V_S haplotype, already reported in 2010 and 2011 in Boa Vista and Pacaraima [8]. The wild-type Na_V_S haplotype is still missing with predominance of the double *kdr* Na_V_R2 (1016Ile + 1534Cys), except in Rorainópolis, where the Na_V_R1 (1016Val + 1534Cys) predominates. We corroborated that Na_V_R2 leads to higher levels of resistance to pyrethroids [14] once homozygote R2R2 insects were only present among the survivors in the bioassays with Boa Vista and Bonfim. The remaining high levels of resistance to pyrethroids even after the substitution by malathion in Roraima may be associated with the high prevalence of domestic use of insecticides, all composed of pyrethroids and easily acquired in local markets, as reported in other Brazilian states [16, 22]. In addition, we cannot neglect the migration of *Ae. aegypti* resistant populations from the neighboring countries and measures adopted against other vector-borne diseases. On the border between Pacaraima (Brazil) and Santa Helena (Venezuela) there is an intense control of malaria where pyrethroids are employed against *Anopheles* even in the urban area, thus also submitting *Ae. aegypti* to this selection pressure. In Bonfim, it is common to find pyrethroid sprays acquired in Lethen City, on the Guyanese side of the border.

We speculate that the *kdr* Na_V_R2 haplotype in *Ae. aegypti* populations in this region must have migrated mostly from Venezuela as it has been detected in Roraima State at least since 2010, when it was either absent or at low frequencies in the neighboring states Amazonas and Pará [8]. Interestingly, Rorainópolis is the only municipality in Roraima with *Ae. albopictus* colonization; however it is encountered in areas on the border with Amazonas State where the species has been recorded since 2015 (CGVS-SESAU-RR). Therefore, it seems that the transition between the biomes, Amazon Forest in the south and the savannah-like “Lavrado” in the north, has been limiting the dispersion of *Ae. aegypti* from Roraima downwards to the Amazonas State. On the other hand, *Ae. albopictus* from Amazonas has not yet invaded the capital Boa Vista in the Lavrado zone.

Besides new promising tools of *Ae. aegypti* control currently being tested in Brazil, such as *Wolbachia*, release of insects carrying a dominant lethal gene (RIDL) and pyriproxyfen autodissemination stations [23–26], insecticides continue to play an important role on a long- term basis in order to rapidly decrease the densities of a target population and consequently, mitigate the cycle of the arboviruses. Therefore, it is of prime importance that a constant surveillance of insecticide resistance be inherent to the chemical control strategy so as to guide authorities about product efficacy.

## Conclusions

We provided evidence that *Ae. aegypti* populations from Roraima were resistant to the pyrethroid deltamethrin in 2016 and 2018, probably due to selection pressure exerted by household use of insecticide sprays. The *kdr* mutations were present under high frequencies and were probably the main mechanism acting in favor of pyrethroid resistance in these populations. Mortality under 90% in bioassays with malathion was observed in some populations in 2018, evidencing that resistance has also been selected to this chemical. *Aedes albopictus* were absent from our collections. These results are important for our understanding of the status of insecticide resistance of *Ae. aegypti* populations from Roraima, and will help improve vector control strategies that may be applied to diverse localities under similar geographical and urban conditions.

## Supplementary information

**Additional file 1: Figure S1.** Knockdown curve to 0.05% deltamethrin. *Aedes aegypti* populations from Boa Vista, Bonfim, Rorainópolis and Pacaraima, collected in 2016 and 2018, additional to the reference lineage Rockefeller. Red dotted line indicates the *Kd*T_50._

## Data Availability

All data generated or analyzed during this study are included in this published article and its additional file. The raw data are available upon request to the corresponding author.

## Abbreviations

DDT: Dichlorodiphenyltrichloroethane
*Na*_*V*_: Voltage-gated sodium channel gene
WHO: World Health Organization
MoH: Ministry of Health
LIRAa: Quick survey of *Aedes aegypti* indexes
IGR: Insect growth regulator
*Kdr*: Knockdown resistance
RR: Resistance ratio
*Kd*T: Time of knockdown
SNP: Single nucleotide polymorphism
95% CI: 95% Confidence interval
