# Supplementary material for: Evaluation of resistance to pyrethroid and organophosphate adulticides and kdr genotyping in Aedes aegypti populations from Roraima, the northernmost Brazilian State
Source: Parasit Vectors. 2020 May 20;13:264. doi: 10.1186/s13071-020-04127-w (PMC7238546; doi:10.1186/s13071-020-04127-w)
Supplement: Supplementary file 1 — Additional file 1: Figure S1. Knockdown curve to 0.05% deltamethrin. Aedes aegypti populations from Boa Vista, Bonfim, Rorainópolis and Pacaraima, collected in 2016 and 2018, additional to the reference lineage Rockefeller. Red dotted line indicates the KdT50. [file 13071_2020_4127_MOESM1_ESM.pdf]

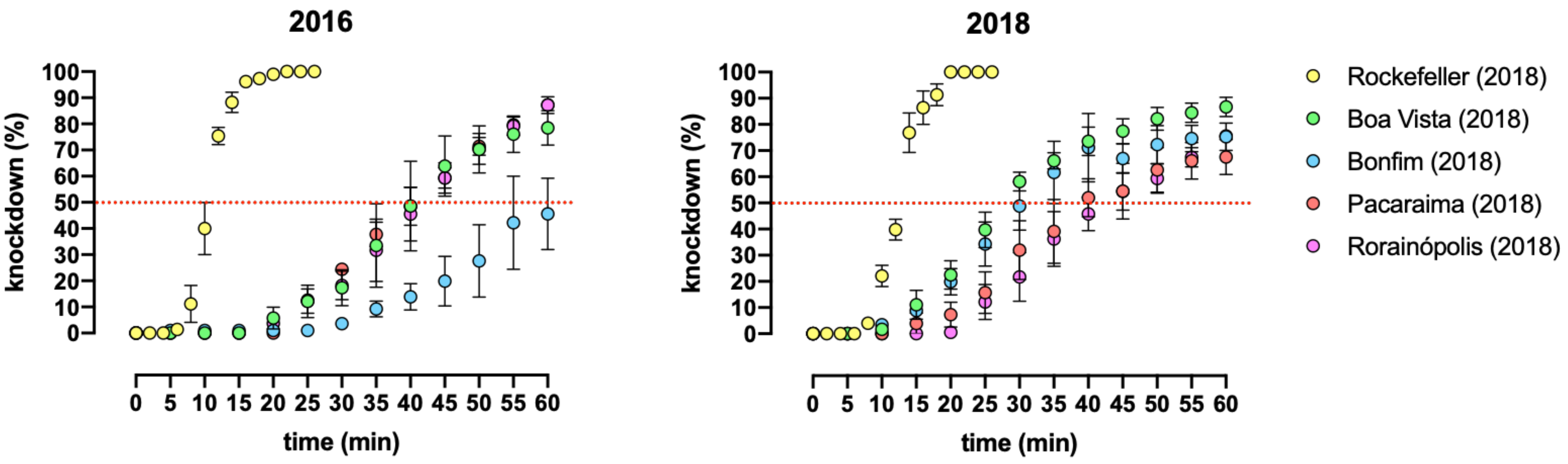

Curve of *knockdown* to deltamethrin 0.05%. *Ae. aegypti* populations from Boa Vista, Bonfim, Rorainópolis and Pacaraima, collected in 2016 and 2018, additional to the reference lineage Rockefeller. Red dotted line indicates the KdT_{50}.
